# Implementing an integrated multidisciplinary telehealth platform: a case study at Taichung Veterans General Hospital

**DOI:** 10.1136/bmjhci-2025-101484

**Published:** 2025-10-15

**Authors:** Pei-Ju Tu, Jin-An Huang, Chi-Sheng Wang, Pi-Shan Hsu, Shi-Yi Lin, Yi-Ting Tsai, Ching-Tsung Chen, Chia-Hua Chu, Hui-Mei Huang, Jiunn-Cherng Lin, Hsin-Ju Tu, Yi-Ju Chen

**Affiliations:** 1 Telehealth Center, Taichung Veterans General Hospital, Taichung, Taiwan; 2 Department of Neurology, Taichung Veterans General Hospital, Taichung, Taiwan; 3 Department of Family Medicine, Taichung Veterans General Hospital, Taichung, Taiwan; 4 Center for Geriatrics and Gerontology, Taichung Veterans General Hospital, Taichung, Taiwan; 5 Division of Metabolism, Department of Internal Medicine, Taichung Veterans General Hospital, Taichung, Taiwan; 6 Computer and Communication Center, Taichung Veterans General Hospital, Taichung, Taiwan; 7 Department of Nursing, Taichung Veterans General Hospital, Taichung, Taiwan; 8 Cardiovascular Center, Taichung Veterans General Hospital, Taichung, Taiwan; 9 Department of Dermatology, Taichung Veterans General Hospital, Taichung, Taiwan

**Keywords:** Continuity of Patient Care, Health Services Accessibility, Telemedicine, Telenursing, Patient-Centered Care

## Abstract

**Objectives:**

To evaluate the impact of implementing a multidisciplinary integrated telehealth platform in central Taiwan on healthcare accessibility, emergency response and chronic disease management.

**Methods:**

We analysed data from 26 institutions within a central Taiwan telehealth network between 2022 and 2024. The study evaluated the use and benefits of teleconsultation, artificial intelligence-assisted ECG monitoring during prehospital ambulance transfers and outcomes in patients with cryptogenic stroke following the platform integration. Satisfaction surveys were performed.

**Results:**

By 2024, more than 300 teleconsultations were performed across 26 partner facilities. Non-emergent referral rates fell from 30% in 2022 to 10% in 2024 following teleconsultations. Emergent stroke teleconsultations allowed thrombolytic therapy within the golden hour in 83% of cases. At-home ECG monitoring helped detect atrial fibrillation in 25% of cryptogenic stroke patients within 2 weeks, ensuring timely recall and initiation of appropriate antiarrhythmic therapy to prevent recurrent stroke. Surveys indicated that 83% of healthcare providers and patients were satisfied with telehealth services.

**Discussion:**

The single-centre study showcases a multidisciplinary integrated telehealth model. However, confounders existed, including changes in the healthcare system, selection bias and technology disparities. Satisfaction data may be biased. The short timeframe precludes long-term analysis, underscoring the need for broader, controlled studies to assess the sustained impact of telehealth.

**Conclusion:**

The integrated telehealth centre model provides a scalable and replicable approach for healthcare delivery. Studies for long-term benefits and outcomes will help improve telehealth models.

**WHAT IS ALREADY KNOWN ON THIS TOPIC**

- Telemedicine has been widely adopted since the COVID-19 pandemic to improve healthcare access.

**WHAT THIS STUDY ADDS**

- This study presents the successful implementation of an integrated multidisciplinary telehealth platform at a single hospital in Taiwan to improve timely emergency management and reduce unnecessary hospital transfers.
- It demonstrates the impact of artificial intelligence (AI)-assisted ECG monitoring on enhancing emergency response in prehospital ambulances and detecting atrial fibrillation in patients with cryptogenic stroke.

**HOW THIS STUDY MIGHT AFFECT RESEARCH, PRACTICE OR POLICY**

- This study provides real-world evidence for the effectiveness of integrated telehealth networks, encouraging further research on AI-driven diagnostics and the long-term impact of telemedicine.
- It promotes the implementation of remote monitoring, 5G ambulance coordination and teleconsultations to enhance healthcare delivery.
- It emphasises the necessity for telemedicine reimbursement, infrastructure development and standardised protocols to guarantee secure and efficient telehealth services worldwide.

## Introduction

 As Taiwan’s population ages and chronic health conditions become more prevalent, the healthcare system faces increased demand for accessible, high-quality medical services. Addressing these challenges, particularly for those in rural and underserved regions, requires a reimagined approach to healthcare delivery. An integrated multidisciplinary telemedicine platform has emerged as a crucial solution, offering the potential to bridge gaps in healthcare access and improve patient outcomes through remote technology.

The telehealth centre at Taichung Veterans General Hospital (TCVGH), established in 2022, serves as a key initiative in Taiwan’s telemedicine landscape, aiming to bring comprehensive, continuous and specialised care to a broad population. Our multidisciplinary telehealth centre seeks to bridge healthcare access gaps by extending specialised medical care to patients in rural areas, reducing the need for travel and addressing urban-rural disparities. It prioritises rapid emergency response by using real-time monitoring and communication networks to streamline critical care and improve outcomes for acute health events. Leveraging advanced technologies like AI and 5G, the centre enhances real-time data analysis, monitoring accuracy and continuity of care, even in remote settings.

This study aims to investigate the impact of implementing an integrated telehealth platform in a central Taiwan network and identify facilitators and barriers to its success.

## Methods

From 2022 to 2024, we evaluated the beneficial impact of implementing the integrated telemedicine platform by analysing data from partnering healthcare institutions within the network. Key outcomes included the number of collaborating institutions, patients receiving telemedicine services such as vital sign monitoring and tele-consultations and the reduction in unnecessary referrals from rural areas. Other outcomes included the proportion of stroke patients receiving timely treatment within the golden hour and increases in subspecialty telemedicine services for rural areas, and satisfaction levels among healthcare providers (HCs) and patients were also analysed. This study has been approved by the ethical review board of Taichung Veterans General Hospital (CG23118A, CE 22440B, CE25005B).

### Organisational structure

**Figure 1:**
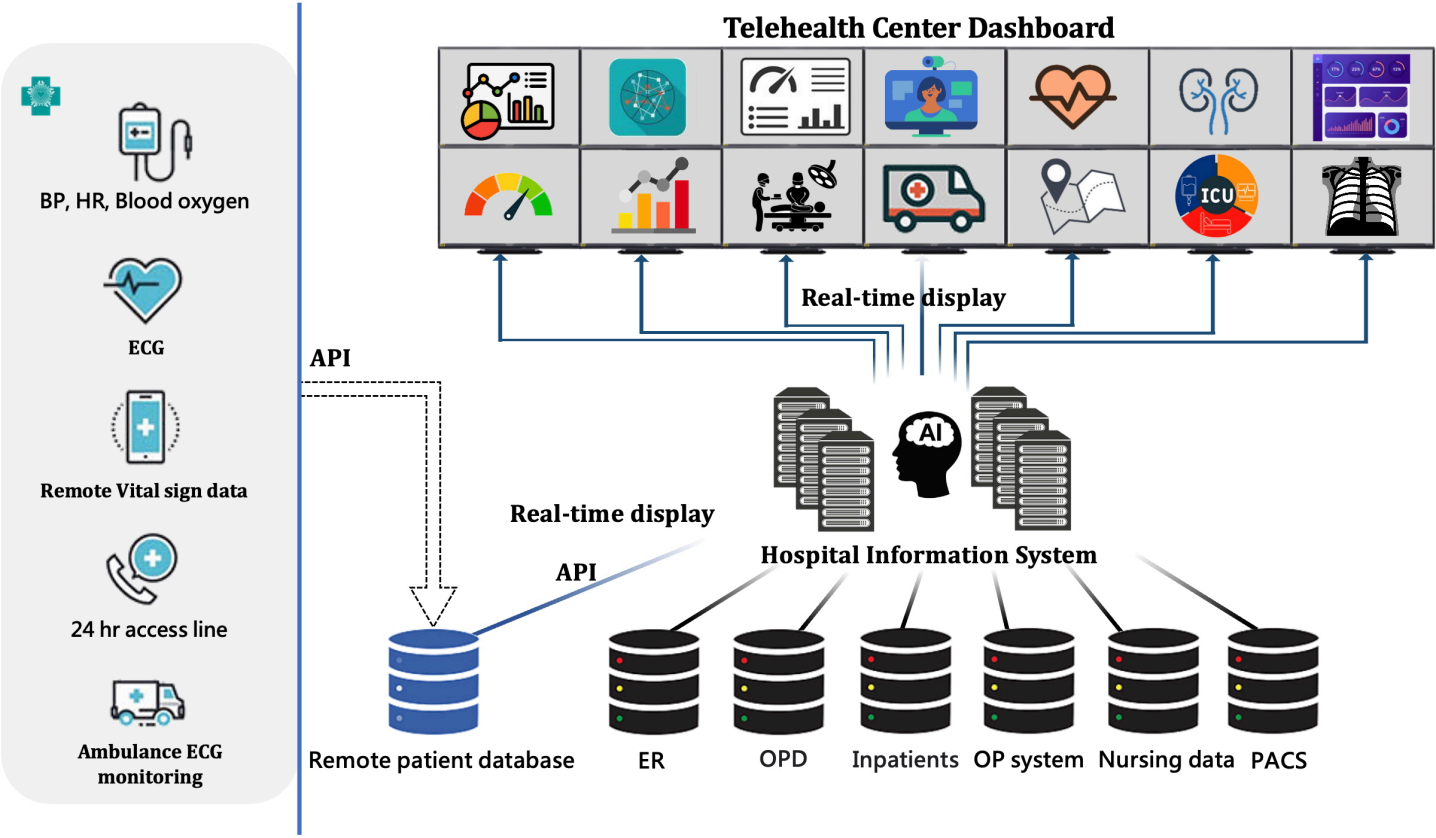
The technical infrastructure of the telehealth system and hospital information system. AI, artificial intelligence; API, application programming interface; BP, blood pressure; ECG, electrocardiogram; ER, emergency room; ICU, intensive care unit; OP, surgical operation; OPD, outpatient department; PACS, picture archiving and communication system.

The telehealth centre is structured to optimise efficiency and ensure comprehensive service coverage, with a focus on addressing acute and chronic care needs. Its organisational framework consisted of one executive leadership team and four working groups. (figure 1) The home care team manages and supports patients in home-based care settings. It includes specialists from family medicine, cardiology, neurology and geriatrics. It provides a robust support system for managing chronic illness by performing remote monitoring and real-time health assessments, aiming to reduce emergency admissions and improve patient quality of life through continuous care. The teleconsultation team supports complex and urgent cases, including experts from various specialties who provide teleconsultation services. Through advanced video consultation platforms, the teleconsultation team assists in procedures such as remote complex surgery support and critical case management, especially in cardiac or neurological emergency cases. The health management and nursing care team encompasses patient education, health assessments and monitoring, with a 24-hour call centre staffed by trained nursing professionals. This team provides continuous support, assisting patients with health queries, monitoring real-time vital signs and ensuring adherence to long-term care plans. The information and engineering team maintains the technological backbone of the telehealth centre. It oversees the functionality and security of telecommunication tools, data storage systems and patient monitoring devices, ensuring seamless and secure data transfer during remote consultations and real-time monitoring sessions. All members of the working groups hold regular weekly meetings and also meet biweekly with the executive leadership team and the hospital superintendent to monitor the progress on various initiatives.

### Technological framework

The technological framework of the Telehealth Centre integrates multiple innovative tools to ensure effective remote patient care and seamless data flow across systems. The centre features a high-resolution web-based video consultation platform equipped with QOCA ATM, QOCA APC platform (Quanta Computer, Taiwan), and AVer MD330 U camera (AVer Information, Taiwan) that enables real-time video consultations, vital signs signals, image transmission and procedure support. The high-speed 5G connectivity supported by Chunghwa Telecom (Taiwan) connectivity enables reliable, high-quality data transmission, which is essential for critical cases that require continuous monitoring and rapid response. For patients at home or in nursing institutions, we employ Taiwan Food and Drug Association-certified Internet of Things (IoT) devices and AI diagnostic models, providing patients with verified and reliable medical equipment to patients.

### Collaboration models and data management

Data privacy and security are key priorities within the centre’s data management protocols. The telehealth centre uses a centralised, secure data-sharing platform to facilitate seamless care transitions and ensure patient information remains accurate and accessible across locations. Our hospital is ISO 27001:2013 certified for information security. The QOCA teleconsultation and healthcare platform, currently in use, operates on our private on-premises cloud. Records of teleconsultations are securely stored on the hospital’s local servers for future reference. Digital data from various IoT devices are securely transmitted to dedicated servers within our hospital’s internal network via encrypted HTTPS. To ensure safe and compliant data handling, our information technology department collaborates with partner companies to integrate their data through controlled Application Programming Interface (API)-based verification. All data linking and storage occur within our hospital information system, which is certified under International Organization of Standardization (ISO) 27001 and adheres to Health Insurance Portability and Accountability Act (HIPAA)-equivalent standards (figure 1). These certifications reflect our commitment to strict cybersecurity protocols and data governance policies that protect patient privacy and ensure system integrity. Both the hospital and system vendors are ISO 27001 certified. Additionally, our 5G network and cloud service partners meet international standards, including ISO 27001, ISO 27017, ISO 27018 and ISO 27701, ensuring robust data security and compliance.

### Satisfaction surveys of teleconsultation

We conducted a cross-sectional survey to estimate the average satisfaction score among users of teleconsultation services. A five-item questionnaire with a five-point grading scale was administered after each teleconsultation session to both HCPs and patients. Each item was rated on a 5-point Likert scale ranging from 1 (very unsatisfied) to 5 (very satisfied), with higher scores indicating greater satisfaction. Subjects rated scale 4 or above were considered satisfactory. The five items included (a) During teleconsultations (remote consultations), is the workflow/operational process convenient? (b) Teleconsultations can help patients (you) and their families (your families) understand medical treatment methods; (c) teleconsultations can encourage family members or caregivers to participate more actively in the medical process; (d) is the process for arranging a hospital transfer (referral) smooth? (e) Does the teleconsultation process (eg, diagnosis, treatment, privacy, information transmission) make you feel safe and trustworthy?

To estimate the mean satisfaction score with a 95% confidence level and a margin of error of ±0.3, the required sample size was calculated using the formula:


$$n={\left(\frac{Z\cdot \sigma }{E}\right)}^{2}$$

where, *Z*=1.96 (for 95% confidence), *σ*=1 (estimated SD for Likert scales) and *E*=0.3 (margin of error). The resulting minimum sample size was approximately 43. To increase reliability and allow subgroup exploration, we aimed to collect responses from at least 100 participants. Descriptive statistics were used to calculate the mean of satisfaction scores.

## Results

### Teleconsultations for networking hospitals in rural regions

Since 2022, we have collaborated with seven hospitals for emergent teleconsultations, including two branch Veterans Hospitals and four affiliated veterans homes in central Taiwan. By 2024, the number of collaborating institutions expanded to 26, accompanied by the establishment of platforms and teleconsultation workflows, including regional hospitals and local health centres in rural areas throughout central Taiwan.

As the hub for the network, we facilitated over 300 teleconsultations for the networking hospitals. Teleconsultation requests from affiliated veterans’ homes increased from 59 visits in 2022 to 269 in 2023 and 258 in 2024. Consultations were conducted for both acute and chronic diseases, assisted by family medicine, other specialties and the emergency services at night. Emergent teleconsultations were mostly for acute ischaemic stroke. These teleconsultations significantly reduced the non-emergent referral rate from 30% in 2022 to 10% in 2023 and 12% in 2024.

### Satisfaction survey for teleconsultation

Of the 276 responders, a total of 230 (83%) expressed satisfaction, including 203 HCPs and seven patients. The average satisfactory score was 4.11 for all respondents. About 10% of HCP respondents were unwilling to use teleconsultation again. Some raised complaints, such as inadequate supporting infrastructure, poor internet connectivity in rural or mountainous regions and the digital divide in learning new IoT devices, often resulting in inefficiency and inconvenience for users. Additionally, telemedicine has been criticised for its timeliness in emergency medical situations. Despite these challenges, most users provide positive feedback, recognising its potential to significantly improve access to appropriate medical care for underserved and remote community residents.

### Advanced telestroke consultation ensured timely treatment.

**Figure 2:**
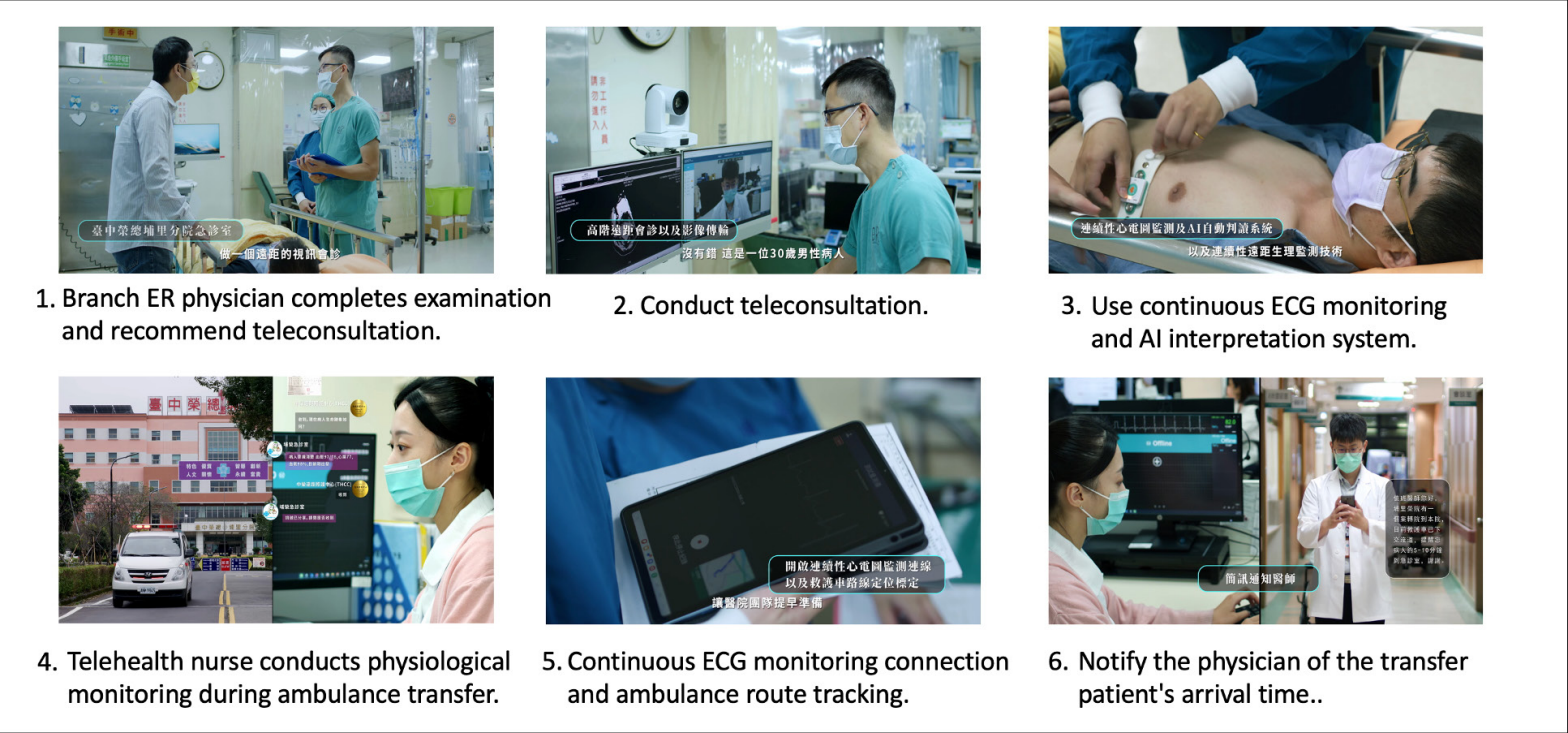
Advanced telestroke consultation using real-time biometric data and image transfer over high-speed internet ensured prompt treatment. AI, artificial intelligence; ECG, electrocardiogram; ER, emergency room.

Emergency telemedicine video consultations increased from 10 cases in 2022 to 24 in 2023 and 21 in 2024. Teleconsultations for acute ischaemic stroke patients ensured timely treatment; 83% received thrombolytic therapy within the golden time from the onset, and 60% completed it within 90 min. For those needing further thrombectomy, 86% received endovascular thrombectomy in 120 min. Following teleconsultation recommendations and subsequent referrals, 83% of critically ill patients transferred to the emergency room stayed for less than 120 min, underscoring the vital role of teleconsultation in improving emergency medical efficiency (figure 2).

### Advanced teleconsultation for complex cardiac catheterisation surgery and high-risk patients

We have also performed advanced telemedicine and surgical consultations for complex surgeries and high-risk patients who require the collaboration of multiple specialists. Our hospital uses high-resolution cameras and advanced imaging capture systems, seamlessly integrating data from vital signs monitors and various sensor technologies, including X-ray, optical coherence tomography systems and intravascular ultrasound devices. By leveraging a 5G network and a video platform, patient vital sign data are transmitted in real-time, enabling faster diagnosis and treatment. Our cardiology experts have successfully managed six cases of refractory arrhythmia, achieving good control in all cases, with one patient achieving a cure.

### Real-time remote transmission of continuous ECG and ambulance localisation using 5G high-speed internet

Until the end of November 2024, we have monitored 454 ambulance transfers from networking hospitals. Since January 2023, the telehealth centre has equipped ambulances with ECG monitors that transmit data via Bluetooth to tablets, which then send real-time ECG signals and GPS locations to the hospital dashboard through a 5G network. Nurses at the telehealth centre monitor ECG signals in real-time, using AI-assisted, colour-coded alerts to identify abnormalities and communicate with the ambulance team to evaluate the patient’s condition. On the patient’s arrival at our emergency department, a fast-track system is initiated, which minimises waiting and reduces delays. This system enhances patients’ safety through continuous monitoring, seamless communication and timely interventions during transfers.

### Continuous ECG monitoring for cryptogenic stroke patients at home

**Figure 3:**
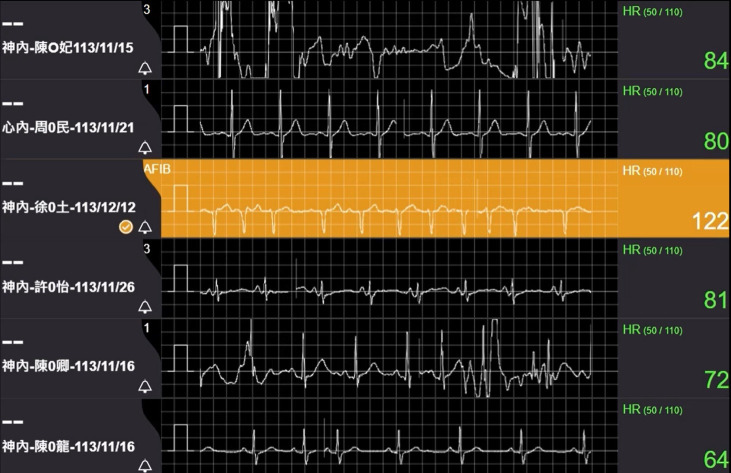
Real-time remote transmission of continuous ECG and ambulance localisation using 5G high-speed internet. Nurses at the Telehealth Centre keep connections and monitor the patients’ biometric data during ambulance transport. The nurses would notify the physician before arrival to follow-up on the patient’s management. AFib, atrial fibrillation; HR, heart rate.

Atrial fibrillation (AFib) has been identified as one of the underlying causes of cryptogenic stroke.^1^ To date, there is no consensus on the effectiveness of continuous ECG monitoring in this context. To improve real-time care, our centre pioneered AI-assisted continuous ECG monitoring to identify arrhythmia patterns, including AFib, ventricular tachycardia, ventricular fibrillation and prolonged pauses, while also monitoring the postdischarge status of stroke patients to provide timely medical support. From November 2023 to November 2024, we used remote real-time ECG monitoring for 68 cryptogenic stroke cases. AFib was successfully detected in 16% of patients within an average of 8.7 days, with an additional 9% identified after 14 days. All patients with identified AFib were recalled for timely intervention, and no recurrent strokes have occurred until now (figure 3).

## Discussion

We demonstrated the successful implementation of the multidisciplinary telehealth platform at TCVGH, which serves as a hub for networking hospitals. Using this innovative system-integrated platform, we have improved consultation efficiency and lowered access barriers. Additionally, this web-based telehealth platform enhanced emergency response and the quality of care, as reflected by the reduced non-emergency referrals and improved patient adherence. The AI-powered monitoring platform enhances diagnostic accuracy and predicts significant arrhythmias in post-stroke patients. Providing timely alerts enables telecare nurses to take proactive measures, reducing the risk of stroke recurrence.

In addition to integrating multiple functions into a unified platform, our centre has also pioneered several innovative approaches in advanced telehealth domains. One of these initiatives involves providing long-term, real-time continuous ECG monitoring for at-risk patients in a home setting. Continuous ECG monitoring enables the early detection of rare but potentially fatal heart rate abnormalities, allowing timely physician intervention to prevent serious cardiac events. AFib is the most recognised factor for cryptogenic stroke. Traditionally, physicians used 14-day Holter ECG recordings to identify high-risk AFib cases; however, the data could only be reviewed when the patient returned to the clinic 2 weeks later, delaying potential diagnosis and intervention.^2^ To enhance real-time care, our centre pioneered continuous ECG monitoring to track the postdischarge status of stroke patients for up to 30 days, identifying an additional proportion of arrhythmia patients and offering timely medical support.

The experience of our integrated telehealth platform is on par with, if not superior to, the telemedicine practices in countries with long-standing expertise in this field. The Cleveland Clinic’s virtual care centre operates 24/7, offering integrated services across multiple specialties including remote intensive care unit monitoring and real-time ambulance telemetry.^3^ Their system has shown improved patient outcomes through coordinated virtual care delivery and rapid specialist access.^4^ Mayo Clinic’s connected care centre has pioneered advanced remote patient monitoring systems that integrate with home health devices and provide virtual emergency services.^5^ In Asia, Singapore General Hospital has implemented a sophisticated telehealth command centre that coordinates virtual specialist rounds, emergency vehicle telemedicine and chronic disease monitoring.^6^ These centres have demonstrated the benefit of integrating telehealth care. However, implementation approaches vary based on regional regulations and infrastructure capabilities.

Telehealth in the European Union (EU) further emphasises the collaboration of cross-border eHealth services. Specialty care integration has been supported by initiatives like the eHealth Digital Service Infrastructure, which comprises 17 partners from 10 European countries, facilitating cross-border health data exchange and projects like e-Mental Health Innovation and Transnational Implementation Platform, which deploys a combined digital and face-to-face mental health programme in North Western Europe. However, issues with interoperability, language barriers, reimbursement and quality control remain.^7^,^9^ Addressing the legal, regulatory and funding barriers to establishing a unified digital health infrastructure and enhancing care accessibility remains a critical next step.^10^

### Limitation

This study has several limitations that should be carefully considered. First, the observational case study at a single centre lacks a control group and randomisation, making it difficult to attribute observed outcomes solely to the telehealth intervention. Potential confounding factors, including the global healthcare system improvements during the study period, might have influenced the results. First, selection bias may occur because the participating institutions and patients were selected based on geographic accessibility and technological readiness, limiting the generalisability of our findings to broader populations. Variability in technological infrastructure and inconsistent internet connectivity in rural and mountainous regions further confound the outcomes. Moreover, differences in digital literacy among HCPs and patients could influence user experiences, satisfaction levels and adoption rates, potentially biasing satisfaction survey results. This survey specifically focused on rural and underserved regions, which may introduce selection bias and limit the generalisability. The use of self-reported satisfaction questionnaires may introduce response biases, such as social desirability or recall bias, which could lead to overinflated satisfaction ratings. Lastly, due to the limited observation period, this study primarily assessed immediate and short-term outcomes rather than long-term health outcomes or detailed cost-effectiveness evaluations. Further studies with longer observation time, broader patient populations and standardised technology frameworks are essential to validate these initial findings and fully understand the telehealth platform’s long-term impact.

## Conclusions

The telehealth centre model offers a scalable and replicable method for global healthcare systems. By combining AI, 5G networks and a multidisciplinary approach, the centre has enhanced patient accessibility, improved emergency response times and set a new standard for telemedicine in chronic disease management and preventive care. We demonstrated how telemedicine can meet the complex needs of an ageing population while addressing healthcare access disparities. Ongoing expansion and technological advancements will enable the centre to further improve healthcare outcomes, establishing a benchmark for future telehealth initiatives worldwide.

## Data Availability

Data are available upon reasonable request.
